# The relationship between balance and visuospatial attention on hemispheric stroke survivors: A study of egocentric and allocentric neural processing

**DOI:** 10.1016/j.nicl.2025.103861

**Published:** 2025-08-09

**Authors:** Shijue Li, Kai Li, Ziyan Huang, Zhenwen Liang, Huaqing Chen, Yongping Zheng, Chuhuai Wang, Qiuhua Yu, Minghui Ding

**Affiliations:** a Department of Rehabilitation Medicine, The First Affiliated Hospital, Sun Yat-sen University, Guangzhou 510080 Guangdong Province, China; b Department of Rehabilitation Medicine, The First Affiliated Hospital of Jinan University, Guangzhou, China; c Department of Biomedical Engineering and Research Institute for Smart Ageing, The Hong Kong Polytechnic University, Hongkong, China

**Keywords:** Balance function, Visuospatial attention, Egocentric reference frame, Allocentric reference frame, Event-related potentials, Stroke

## Abstract

**Background:**

Impaired balance and visuospatial attention are well-documented sequelae of stroke. However, the interplay between balance function and visuospatial attention, particularly within egocentric and allocentric reference frames, remains poorly understood.

**Objective:**

This study aimed to elucidate the relationship between balance and visuospatial attention in stroke survivors with left- and right-hemisphere lesions and to investigate the underlying neural mechanisms.

**Methods:**

Seventeen patients with right-hemisphere stroke, sixteen with left-hemisphere stroke, and eighteen age-matched healthy controls participated in this study. Balance function was evaluated using Prokin, while visuospatial attention was assessed through tasks involving egocentric and allocentric reference frames. In addition, event-related potentials of the P1, N1, and P2 components were measured during the attention tasks.

**Results:**

Patients with left-hemisphere stroke exhibited superior balance and visuospatial attention performance compared to those with right-hemisphere stroke. Balance function was positively correlated with both egocentric and allocentric visuospatial attention performance in left-hemisphere stroke survivors. Attenuated P1 amplitudes and enhanced P2 amplitudes were observed during allocentric processing in right-hemisphere stroke survivors. The P2 amplitude at the O2 electrode was positively associated with medio-lateral velocity, ellipse area, and perimeter during balance tasks in left-hemisphere stroke survivors.

**Conclusions:**

Balance function is closely linked to selective attention and categorization processes in allocentric visuospatial tasks, particularly in patients with left-hemisphere stroke, suggesting that the right hemisphere may play an important role in mediating balance and visuospatial attention functions in the patients with mild to moderate stroke.

## Highlights

• The visuospatial attention capacity is impaired in right hemisphere stroke patients.
• Cerebral hemispheres show different roles of allocentric and egocentric processing.
• Right hemisphere stroke patients show an adaptive strategy in allocentric processing.
• The relation between balance and visuospatial attention is hemispherically dependent.

## 1 Introduction

Stroke is the leading cause of mortality and disability in China, imposing a significant burden on public health systems (Wu et al., 2019). Among the diverse clinical sequelae of stroke, balance dysfunction is a pervasive and debilitating symptom. This impairment not only limits patients' mobility but also increases the risk of falls, profoundly disrupting their daily activities and overall quality of life (Khan et al., 2022, Dantas et al., 2023). While balance control is traditionally associated with motor and sensory systems, recent studies have shown the intricate interplay between balance control and cognitive functions such as memory, executive function, and visuospatial attention (Bergqvist et al., 2023, Mihara et al., 2021, Peters et al., 2015, Yu et al., 2021). These findings underscore the importance of understanding the cognitive underpinnings of balance control to inform targeted rehabilitation strategies and improve patients’ outcomes.

Visuospatial attention, a critical cognitive function, enables individuals to perceive the spatial location of objects relative to their own body and to discern the spatial relationships among multiple objects (Höhler et al., 2021). This ability is organized within two distinct reference frames: egocentric and allocentric (Lithfous et al., 2014). The egocentric reference frame involves a self-centered perspective, encoding spatial information relative to the observer’s own position, whereas the allocentric reference frame adopts an object- or stimulus-centered perspective, relying on external spatial cues independent of the observer’s position. In stroke survivors, disruptions in visuospatial attention are common and have been shown to impair motor planning for voluntary, goal-directed movements, thereby adversely affecting balance and gait (Peters et al., 2015). For example, visuospatial attention has been reported to be positively correlated with long-term improvements in balance function, with its impact persisting up to one year post-stroke (Bergqvist et al., 2023). Furthermore, visuospatial attention assessed two months after stroke has been found to predict balance capacity even two years later (Robertson et al., 1997), highlighting its critical role in post-stroke rehabilitation. Despite these findings, limited research has explored the distinct contributions of egocentric and allocentric reference frames to the relationship between visuospatial attention and balance function in stroke survivors.

Event-related potentials (ERPs) are time-locked brain responses that reflect neural activity associated with specific sensory, cognitive, or motor events (Luck et al., 2000). ERPs have been shown to differ between allocentric and egocentric conditions, providing valuable insights into visuospatial attention processes. The P1 and N1 components at parietal sites are thought to index attentional allocation and discrimination process respectively. Additionally, the P2 component, often observed in visuospatial attention tasks, has been linked to selective attention and categorization (Lithfous et al., Sep 2014, Lithfous et al., 2013). Notably, impaired allocentric performance in older adults is characterized by increased P2 amplitude and prolonged P2 latency at parietal regions compared to younger individuals, reflecting age-related cognitive resource demands. Functional imaging studies further reveal that allocentric reference frame processing engages a broader attentional cerebral network than egocentric processing, suggesting that allocentric tasks require greater cognitive resources (Wilson et al., 2005, Iachini et al., 2023). These findings highlight a dissociation in the neural mechanisms underlying allocentric and egocentric spatial representations. Given this neural dissociation and the sensitivity of ERPs to attentional processing, ERP analysis offers a promising approach to investigate how stroke impacts attentional mechanisms in allocentric and egocentric reference frames.

Previous study has shown that selective and focused attention function are differentially affected by the side of brain lesions, with varying degrees of impairment observed (Iachini et al., 2023). Neuroimaging studies indicate that attentional processes predominantly involve a right-hemisphere cortical and subcortical network, particularly the parietal and occipital cortices, as well as the thalamus (Sturm and Willmes, 2001, Sheynikhovich et al., 2009, Fan et al., 2005). Moreover, patients with right-hemisphere lesions often exhibit more pronounced attention deficits compared to those with left-hemisphere lesions (Spaccavento et al., 2019). These findings suggest that the side of the lesion may play a critical role in determining the degree of impairment in visuospatial attention functions.

Building on this evidence, the present study aimed to investigate the relationship between balance and visuospatial attention functions, specifically within the context of egocentric and allocentric reference frames, in stroke survivors with left- and right-hemisphere lesions. By employing ERPs, we sought to elucidate the underlying neural mechanisms associated with these functions. We hypothesized that stroke survivors with left-hemisphere lesions would demonstrate better balance and visuospatial attention functions compared to those with right-hemisphere lesions. Additionally, we proposed that balance function would be positively correlated with egocentric and allocentric visuospatial attention in left-hemisphere stroke survivors. Finally, we anticipated that P1 and P2 amplitudes would be changed during visuospatial attention in right-hemisphere stroke survivors. And P2 amplitude might be associated balance performance in left-hemisphere stroke survivors.

## 2 Methods

### 2.1 Participants

This study recruited 17 right-hemisphere stroke survivors (RH group), 16 left-hemisphere stroke survivors (LH group) and 18 matched healthy controls (HC group) from the Department of Rehabilitation Medicine at The First Affiliated Hospital of Sun Yat-sen University, between October 2022 and October 2023. Individuals post-stroke met the following inclusion criteria: (1) a confirmed diagnosis of ischemic or hemorrhagic stroke based on computed tomography or magnetic resonance imaging; (2) post-stroke duration between 1 and 6 months; (3) age between 35 and 75 years; (4) the ability to understand and cooperate with the visuospatial attention task, including using the unaffected hand to operate a keyboard; (5) the ability to stand unassisted for more than 30 s (Lopes et al., 2015); (6) right-handed before the stroke and (7) compliance with the experimental procedures. Exclusion criteria included the presence of severe aphasia (the Aphasia Severity Rating Scale of the Boston Diagnostic Aphasia Examination < 3) (Glize et al., 2017), unilateral neglect (The Catherine Bergego Scale and The Behavioural Inattention Test Azouvi (2017)), or hemianopia. The inclusion criteria for healthy controls were right-handed and had matched age. The study protocol was approved by the Clinical Research Ethics Committee of The First Affiliated Hospital of Sun Yat-sen University (Approval No. [2022]669). All participants provided informed consent.

### 2.2 Balance function test

Balance function was assessed using the Prokin 254P System (Tecnobody, Italy). Participants stood barefoot on a static platform with their eyes open for 30 s during each trial, repeating the task three times. Four outcome parameters were measured: perimeter, ellipse area, and sway velocity in the anterior-posterior and medio-lateral directions (Lopes et al., 2015). Perimeter was calculated as the difference between the maximum and minimum coordinates of the center of pressure (COP) in each direction and was expressed in millimeters (mm). The ellipse area encompassing the complete COP trajectory was calculated by multiplying anterior-posterior and mediolateral amplitudes and was expressed in square millimeters (mm^2^). Sway velocity was calculated by dividing the total length of the COP trajectory by the duration of the recording and expressed in millimeters per second (mm/s) (Lopes et al., 2015). COP displacement is regarded as the most reliable parameter for assessing balance function (Roerdink et al., 2011).The mean values of these parameters across trials were calculated and used for data analysis. Higher values indicated poorer balance function.

### 2.3 Visuospatial attention task

**Fig. 1:**
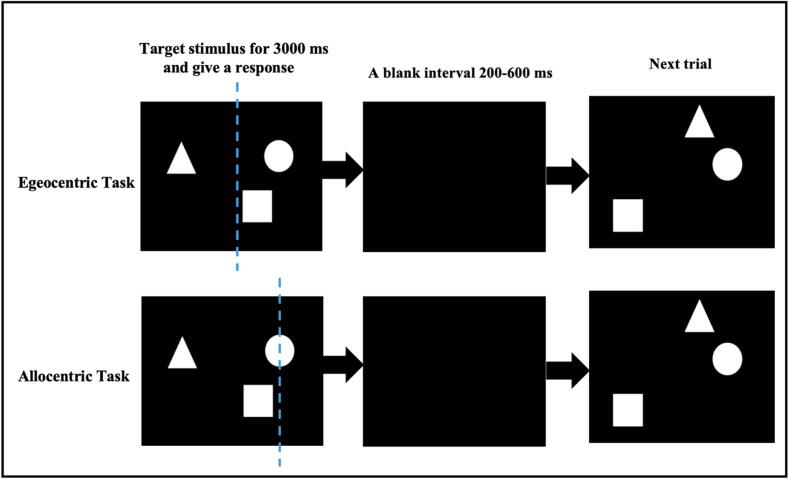
Example of stimuli used in egocentric and allocentric spatial conditions. In the egocentric task, the blue dotted represents the midline of screen. In the allocentric task, the blue dotted represents the midline of circle. (For interpretation of the references to colour in this figure legend, the reader is referred to the web version of this article.)

Participants completed the task in a dimly lit, sound-proof, electrically isolated chamber to eliminate external distractions. The visuospatial attention task employed a block design with two reference frame conditions: egocentric and allocentric. Stimuli consisted of geometric shapes, including circles, squares, and triangles, displayed randomly against a black background (Fig. 1). Shapes were presented in eccentric positions, with each target stimulus displayed for 3000 ms, followed by a blank screen with a randomized interval (200–600 ms). Stimuli were presented using E-Prime 2.0 software. In the egocentric reference frame condition, participants judged whether a triangle was positioned to the left or right relative to their own midline. In the allocentric reference frame condition, participants determined whether a triangle was located to the left or right of a circle. Once the stimulus was displayed in the screen, responses were made using the index finger of the unaffected hand, pressing the “V” key for “left” and the “B” key for “right.” Each condition consisted of 50 trials (10 practice and 40 formal experimental trials). Reaction time (RT) and accuracy rate (ACC) were recorded for each trial. To account for the speed-accuracy trade-off, the inverse efficiency score (IES) was calculated as IES = RT/ACC (Zou et al., 2017, Yu et al., 2022), with smaller IES values indicating better visuospatial attention performance.

### 2.4 ERP data acquisition

Electroencephalography (EEG) signals were recorded during the visuospatial attention task using a 32-channel active electrode cap (Brain Products, Germany). Electrode placement followed the standard 10–20 system. Data were sampled at 1000 Hz, and electrode impedance was maintained below 10 kΩ throughout the recording to ensure data quality.

### 2.5 EEG preprocessing

Offline EEG data were preprocessed using BrainVision Analyzer 2.1 (Brain Products). EEG signals were re-referenced to the common average reference. Eye movement artifacts were removed using Independent Component Analysis (ICA) as part of the standard preprocessing pipeline. Signals were filtered with a bandpass filter (0.1–30 Hz) and a 50 Hz notch filter to remove power line noise. EEG data were segmented into epochs time-locked to the onset of the target stimulus, spanning from 200 ms before to 1000 ms after stimulus onset. Baseline correction was applied using the pre-stimulus interval. Epochs with amplitudes exceeding ± 100 µV or containing incorrect responses were excluded from further analysis. Time windows for ERP components were defined as follows: P1 (80–150 ms), N1 (150–250 ms), and P2 (280–380 ms) (Lithfous et al., 2014). Grand average waveforms for these components were analyzed at P7, P8, O1 and O2 electrode sites. The P7 and P8 electrode sites were for parietal region. The O1 and O2 electrode sites were for occipital region. The regions were chosen because they are associated with visual input and they have been selected for data analysis in most previous studies of visuospatial attention (Li et al., 2023, Dombrowe and Hilgetag, 2014, Bonacci et al., 2020, Bacigalupo and Luck, 2019). Mean amplitudes for each component were calculated for each individual within the specified time windows.

### 2.6 Data collection procedure

At the beginning, all participants completed some cognitive function assessment, including the Montreal Cognitive Assessment (MoCA) to evaluate overall cognitive function (Nasreddine et al., 2005) and the Trail Making Test (TMT) to test visual scanning, visual attention and mental flexibility (Llinàs-Reglà et al., 2017). The TMT materials consisted of two parts (TMT-A and TMT-B). The TMT-A required participants to sequentially connect numbers 1–25 randomly dispersed in circles, while the TMT-B involved alternating between numbers 1–13 and letters A–L. Both tasks were scored by completion time (in seconds). Following these assessments, participants underwent the balance function test and visuospatial attention task.

### 2.7 Data analysis

Statistical analyses were performed using SPSS 26.0, with an alpha level set at 0.05. Categorical variables were analyzed using chi-square tests. For continuous variables, normality was assessed using the Shapiro-Wilk test. If data were normally distributed, differences in behavioral and ERP data (mean amplitudes of P1, N1, and P2) among the three groups (RH, LH, HC) at each electrode (P7, P8, O1, O2) in egocentric and allocentric conditions were analyzed using one-way ANOVA (Li et al., 2023). Post hoc pairwise comparisons with the Tukey test were applied when significant group main effects were observed. We explored the relationship between NIHSS scores with the behavioural and EEG results by using Pearson correlation analysis. When the relationship is significant, we further explored the differences in RH and LH groups with the covariate of NIHSS scores in the behavioural and EEG outcome variables. Pearson correlation analyses were conducted to explore the relationship between balance performance and behavioral outcomes of visuospatial attention task (IES) with FDR correction, the relationships between balance performance and ERP measures of visuospatial attention task for each group. Analyses for egocentric and allocentric reference frames were conducted separately to delineate spatial encoding differences.

## 3 Results

### 3.1 Demographic outcomes

There were no significant differences in demographic and clinical characteristics among the three groups (Table 1, *P* > 0.05). In cognitive function assessments, there were significant differences in MoCA and TMT-A among the three groups (F = 3.300, *P* = 0.045; F = 4.708, *P* = 0.014). Post hoc comparisons revealed that LH and RH groups exhibited lower MoCA scores than HC group (*P* = 0.026 and *P* = 0.040, respectively). Additionally, LH and HC groups required significantly shorter time to complete the TMT-A test compared to RH group (*P* = 0.022 and *P* = 0.006, respectively). Detailed cognitive function assessment results are provided in Supplemental Table 1.

**Table 1:** Demographic and clinical characteristics of three groups.

	RH	LH	HC	F/t/$x^{2}$	*P* value
	n = 17	n = 16	n = 18		
Age,year (mean ± SD)	54.41 ± 14.66	47.44 ± 16.40	48.94 ± 9.07	1.220	0.304
Sex (male/female)	10/7	12/4	11/7	1.102	0.576
Onset time, month (mean ± SD)	2.45 ± 2.05	3.00 ± 2.42	/	−0.706	0.483
NIHSS (mean ± SD)	2.18 ± 1.78	2.50 ± 1.71	/	−0.532	0.319
Stroke type			/	1.411	0.398
·Hemorrhagic	5	2			
·Ischemic	12	14			
Stroke Location			/	0.170	0.919
·Basal ganglia	12	12			
·Thalamus	2	2			
·Frontal lobe	3	2			
Lesion size, mm (mean ± SD)	14.43 ± 12.10	12.06 ± 11.65	/	0.572	0.572
Lower extremity Brunnstrom stage	4.71 ± 0.69	4.94 ± 0.77	/	−0.912	0.369

**P < 0.01. * *P* < 0.05.

### 3.2 Balance function test

**Fig. 2:**
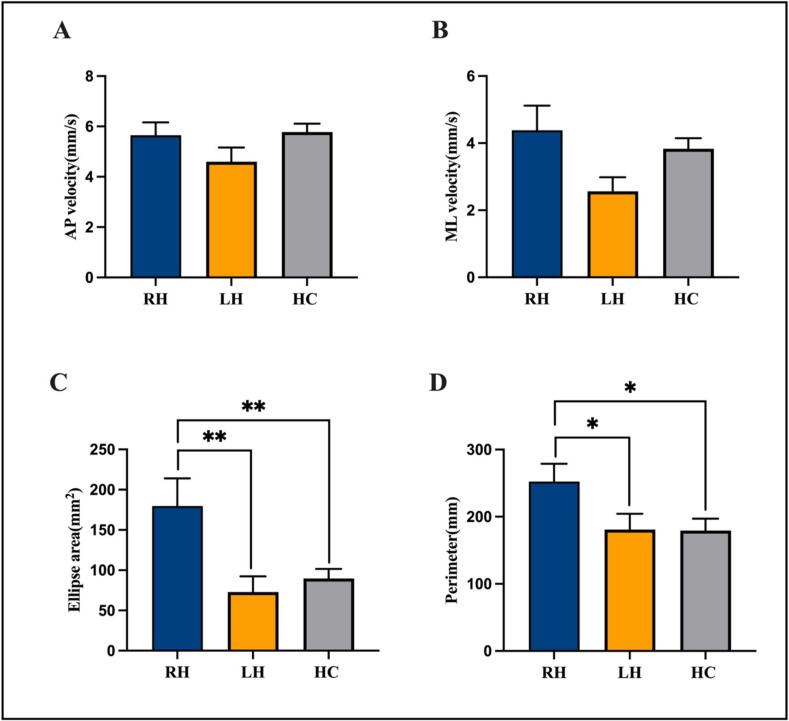
Comparison of anterior-posterior velocity, medium-lateral velocity, ellipse area and perimeter in the three groups. Abbreviation: AP, anterior-posterior; ML, medium-lateral. **P < 0.01. *P < 0.05. Each bar and error bar indicate the mean value and standard error.

In the stroke group, significant correlation was observed between NIHSS scores and ellipse area (r = 0.361, *P* = 0.039). Results from the balance function test are presented in Fig. 2 and Supplemental Table 2. There were no significant differences in anterior-posterior and medio-lateral sway velocity among the three groups (F = 1.827, *P* = 0.172; F = 3.130, *P* = 0.053). There were significant differences in ellipse area, and perimeter among the three groups (F = 5.838, *P* = 0.005; F = 3.357, *P* = 0.043). Post hoc comparisons revealed that LH group showed significantly smaller ellipse area, and perimeter compared to RH group (*P* = 0.003, *P* = 0.034). By the adjustment of NIHSS, the between group differences (LH vs. RH groups) for ellipse area remained significant (*P* = 0.015). Additionally, HC group exhibited a smaller ellipse area and perimeter compared to RH group (*P* = 0.009 and *P* = 0.026, respectively). These findings suggested that LH group experienced better balance performance overall.

### 3.3 Visuospatial attention task

**Fig. 3:**
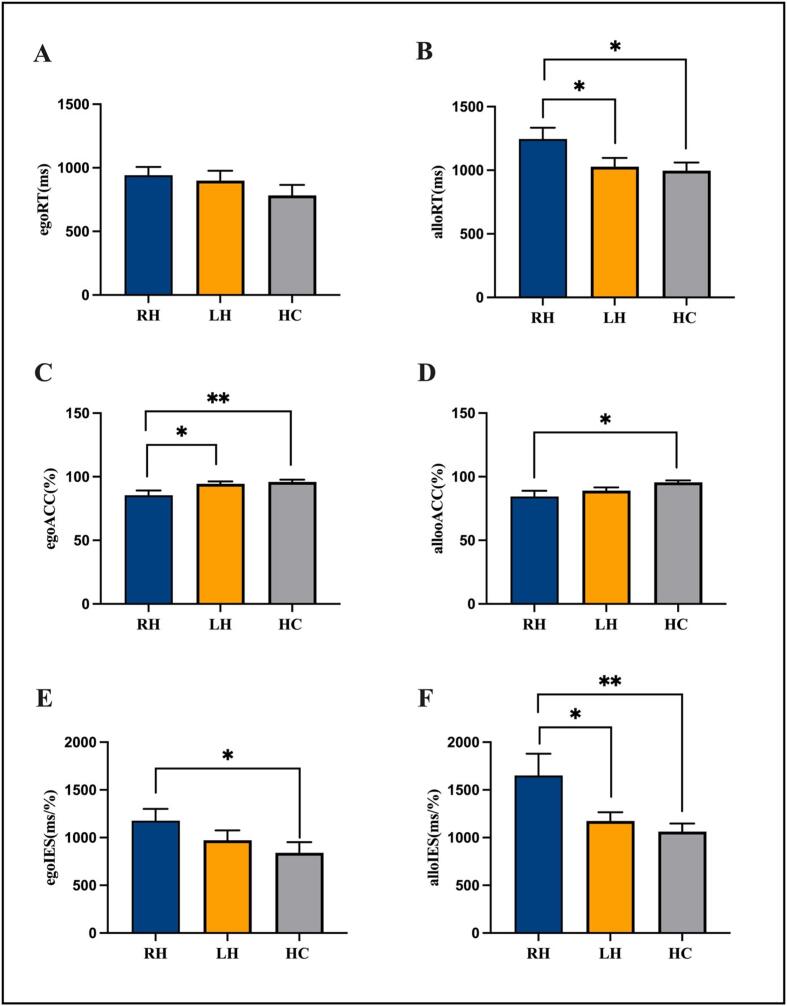
The accuracy rate and response time of visuospatial attention task among the three groups. Abbreviation: EgoRT, egocentric reaction time; EgoACC, egocentric accuracy rate; EgoIES, egocentric inverse efficiency score; AlloRT, allocentric reaction time; AlloACC, allocentric accuracy rate; AlloIES, allocentric inverse efficiency score. **P < 0.01. *P < 0.05. Each bar and error bar indicate the average and standard error.

In the stroke group, no significant correlation was observed between NIHSS scores and visuospatial performance (*P* > 0.05). Thus, NIHSS was not included as a covariate in the analysis of visuospatial outcome differences between RH and LH groups. Behavioral results for the visuospatial attention task are shown in Fig. 3 and Supplemental Table 3. In the egocentric condition, there were no significant differences in reaction time and IES among the three groups (F = 1.213, *P* = 0.306; F = 2.291, *P* = 1.112). There was a significant difference in accuracy among the three groups (F = 4.914, *P* = 0.011). Post hoc comparisons revealed that LH and HC groups had significantly higher accuracy than RH group (*P* = 0.019 and *P* = 0.005, respectively), with no significant difference in accuracy between LH and HC groups (*P* = 0.679). RH group displayed a significantly higher IES compared to HC group (*P* = 0.038), indicating poorer attentional efficiency. In the allocentric condition, there were significant differences in reaction time, accuracy and IES among the three groups (F = 3.293, *P* = 0.046; F = 3.348, *P* = 0.044; F = 4.403, *P* = 0.018). Post hoc comparisons revealed that LH and HC groups showed shorter reaction time than RH group (*P* = 0.049 and *P* = 0.021, respectively), while HC group responded more accurately than RH group (*P* = 0.013). LH and HC groups also exhibited a significantly lower IES compared to RH group (*P* = 0.032 and *P* = 0.007, respectively).

### 3.4 EEG results

**Fig. 4:**
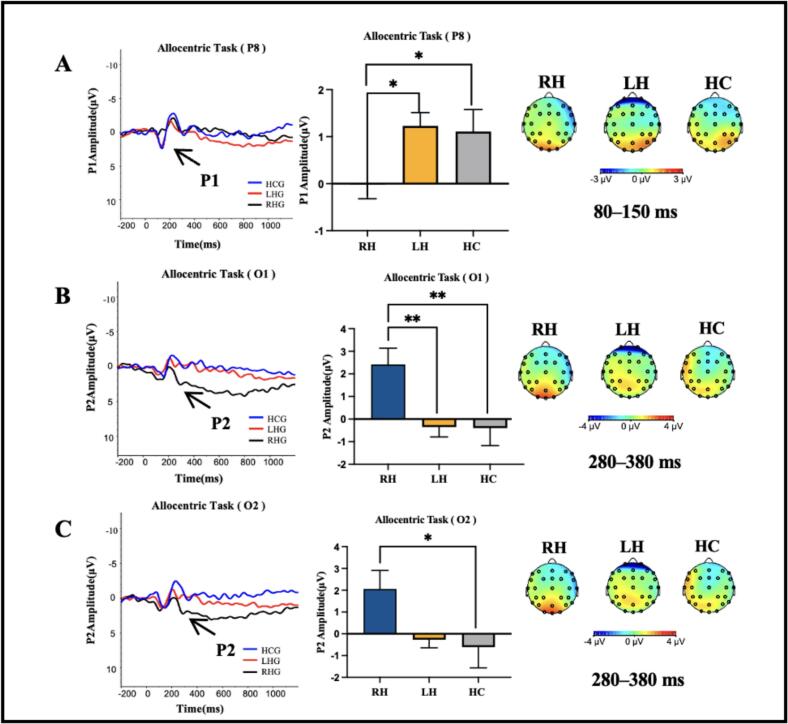
The ERP responses in the egocentric and allocentric condition among the three groups, including waveforms, histogram and topography of the ERPs. **P < 0.01. *P < 0.05. Each bar and error bar indicate the average and standard error.

EEG data from two left hemispheric individual post-stroke were excluded due to poor quality, as more than 50 % of their epochs were rejected. The two participants were removed from behavioural analyses. In the egocentric condition, no significant differences in P1, N1 and P2 amplitude were found at any electrode site among the three groups (*P* > 0.05). In the allocentric condition, there was a significant difference in P1 amplitude at the P8 electrode among the three groups (F = 3.404, *P* = 0.042). Post hoc comparisons revealed that LH and HC groups exhibited a significantly higher P1 amplitude at the P8 electrode compared to the RH group (*P* = 0.027 and *P* = 0.033, respectively). No significant differences in N1 amplitude were found at any electrode site among the three groups (*P* > 0.05). There were significant differences in P2 amplitude at the O1 and O2 electrodes among the three groups (F = 5.553, *P* = 0.007; F = 3.309, *P* = 0.045). Post hoc comparisons revealed that LH and HC groups demonstrated a significantly lower P2 amplitude at the O1 electrode compared to RH group (*P* = 0.009 and *P* = 0.005, respectively) and HC group demonstrated a significantly lower P2 amplitude at the O2 electrode compared to RH group (*P* = 0.020). As shown in Fig. 4, these results suggested a distinct neural processing pattern among the three groups during egocentric and allocentric condition.

In the stroke group, significant correlations were identified between NIHSS rating and P1, N1 and P2 amplitudes at the P7 electrode in the allocentric condition (r = 0.523, *P* = 0.003; r = 0.473, *P* = 0.007; r = 0.415, *P* = 0.020). There were no significant differences between LH and RH groups in P1, N1 and P2 amplitudes at the P7 electrode in the allocentric condition by adjusting the covariate of NHISS scores (F = 0.153, *P* = 0.698; F = 0.691, *P* = 0.413; F = 1.614, *P* = 0.214).

### 3.5 Correlation analysis

**Fig. 5:**
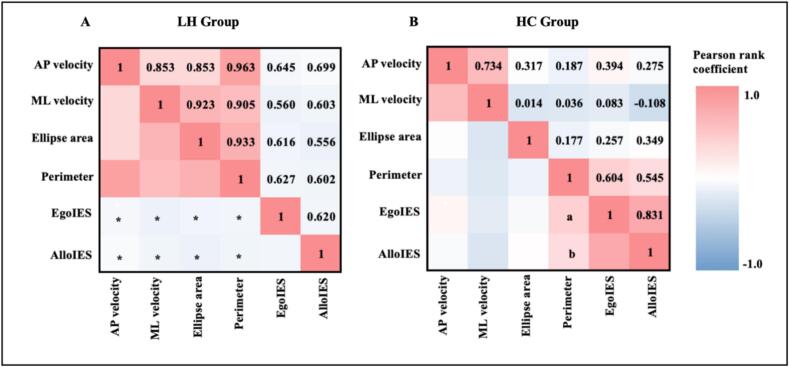
Pearson rank correlation coefficient between balance parameter and the behavioral data in visuospatial attention task in the LH and HC groups. Abbreviation: AP, anterior-posterior; ML, medium-lateral; EgoIES, egocentric inverse efficiency score; AlloIES, allocentric inverse efficiency score. ^a^*P* = 0.008, ^a^*P*_FDR_ = 0.064, ^b^*P* = 0.019, ^b^*P*_FDR_ = 0.076, **P < 0.01, *P < 0.05.

**Fig. 6:**
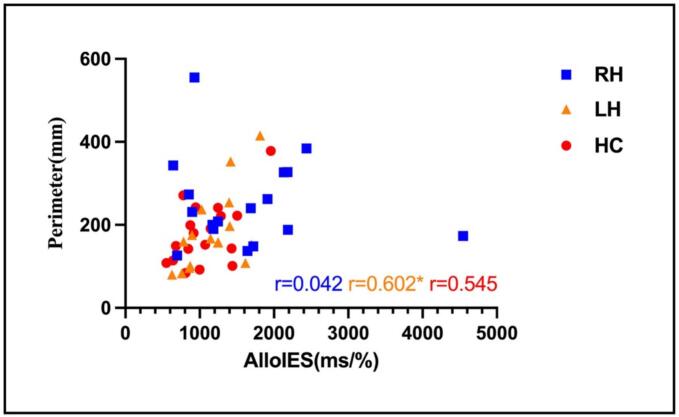
Relationship between perimeter and allocentric IES among the three groups. Abbreviation: AlloIES, allocentric inverse efficiency score. ** *P* < 0.01, * *P* < 0.05.

In LH group, significant correlations were identified between balance performance and visuospatial attention measures in both egocentric and allocentric conditions. For the egocentric condition, anterior-posterior velocity, medio-lateral velocity, ellipse area, and perimeter were positively correlated with IES (r = 0.645, *P* = 0.007, *P*_FDR_ = 0.0187; r = 0.560, *P* = 0.024, *P*_FDR_ = 0.025; r = 0.616, *P* = 0.011, *P*_FDR_ = 0.019; r = 0.627, *P* = 0.009, *P*_FDR_ = 0.019). In the allocentric condition, similar correlations were observed. Anterior-posterior velocity, medio-lateral velocity, ellipse area and perimeter were positively associated with IES (r = 0.699, *P* = 0.003, *P*_FDR_ = 0.019; r = 0.603, *P* = 0.013, *P*_FDR_ = 0.019; r = 0.556, *P* = 0.025, *P*_FDR_ = 0.025; r = 0.602, *P* = 0.014, *P*_FDR_ = 0.019). In RH and HC groups, no significant correlations were observed between balance performance and visuospatial attention measures (*P* > 0.05). The correlation results of LH and HC groups were presented in the Fig. 5. Fig. 6 displayed the relationship between perimeter and allocentric IES for three groups. One outlier of Subjects (AlloIES = 4546.54 ms%) in RH group was excluded from the statistical analysis due to AlloIES larger than 3 standard deviations.

Regarding ERP and balance correlations, the P2 amplitude at the O2 electrode was positively correlated with medio-lateral velocity (r = 0.535, *P* = 0.049), ellipse area (r = 0.667, *P* = 0.009), and perimeter (r = 0.580, *P* = 0.030) in LH group. These correlations were not observed in RH and HC groups, indicating potential lateralized neural mechanisms in visuospatial attention and balance. Detailed results were shown in Supplemental Table 4.

## 4 Discussion

The present study revealed that LH group exhibited superior balance function and enhanced egocentric and allocentric spatial attention capacities compared to RH group. In the LH group, balance function was positively correlated with performance in both egocentric and allocentric conditions. ERP results showed that P1 amplitude at the P8 electrode was lower in the RH group compared to the LH and HC groups during the allocentric reference frame task. Additionally, P2 amplitude at the O1 and O2 electrodes was greater in the RH group than in the LH and HC groups under the allocentric condition. Significant correlations between ERP measures and balance performance were exclusive to the LH group. These findings underscore the distinct neural and functional roles of hemispheric specialization in integrating spatial attention and balance function, providing important insights into lateralized brain mechanisms that could inform targeted interventions or rehabilitation strategies.

Our study revealed that LH and HC groups exhibited better balance performance compared to RH group, aligning with findings from previous studies(Lopes et al., 2015, Frenkel-Toledo et al., 2021, de Sèze et al., 2001). These results support the well-documented role of the right hemisphere in postural balance, as evidenced by poorer balance function in right-hemispheric stroke survivors compared to those with left-hemispheric stroke (de Sèze et al., 2001). There was no significance between LH and HC groups in balance performance. These findings support the involvement of the right hemisphere in maintaining balance while highlighting the possibility of hemispheric-specific compensatory adaptations in LH stroke survivors.

Our findings indicated that LH group exhibited superior performance compared to RH group under both visuospatial reference frame conditions. Additionally, no significant differences in visuospatial attention ability were observed between LH and HC groups. These results align with previous studies (Spaccavento et al., 2019, Zuanazzi and Cattaneo, 2017), supporting the lateralized roles of the cerebral hemispheres in spatial attention processing. Previous hemispatial theory is that the right hemisphere is responsible for attending to both left and right hemisfileds, whereas the left hemisphere primarily attends to the right hemifield. The observed differences in visuospatial attention abilities between the two hemispheric groups may be attributed to the asymmetry in the reception and processing of spatial information by the brain. The right hemisphere is thought to process a greater proportion of visual input, integrating information from both visual fields. Following a lesion in the left hemisphere, the intact right hemisphere may compensate for processing spatial information bilaterally, thereby mitigating the functional impact of the lesion (Zhou et al., 2012, Corbetta and Shulman, 2002, Hoff et al., 2007). Conversely, damage to the right hemisphere disrupts this critical integrative function, leading to poorer visuospatial attention performance. These findings highlight the critical role of the right hemisphere in visuospatial processing and suggest that its compensatory capacity is essential for maintaining spatial attention abilities, particularly following left hemispheric damage.

There was no significant between-group difference in P1 amplitude at any electrode during egocentric processing. Previous studies suggested egocentric processing could elicit occipital early component P1, reflecting top-down attention modulation (Bayer et al., 2017, Lithfous et al., Sep 2014). While RH patients showed reduced egocentric task accuracy compared to LH patients, the absence of corresponding P1 amplitude differences may reflect limited statistical power to detect subtle neural effects given our sample size. We observed that the P1 amplitude at the P8 electrode (parietal lobe) was significantly lower in the RH group compared to the LH and HC groups during the allocentric reference frame task, This finding supports the notion that the P1 component was first evoked brain response reflecting the sensory processing in the visual cortex and the allocation of attentional resources to incoming visual stimuli (Spironelli et al., 2019). The reduced P1 amplitude in the affected hemisphere suggests deficits in both visual sensory processing and attention allocation. The right parietal lobe is particularly critical for initial allocentric processing (Wilson et al., 2005), and the observed reduction in P1 amplitude in the RH group underscores impaired allocation of attentional resources to visual stimuli. This impairment may stem from damage to the attentional networks, specifically the dorsal and ventral attention networks, which are primarily supported by the right hemisphere (Baldassarre et al., 2016, Ruotolo et al., 2006). In contrast, we found that the P2 amplitude at the O1 and O2 electrodes (occipital lobes) was significantly greater in the RH group compared to the LH and HC groups during the allocentric reference frame task. The P2 component is associated with selective attention and categorization processes, reflecting the spatial attention required for stimulus localization within reference frames (Lithfous et al., Sep 2014, Philips and Takeda, 2009). The enhanced P2 amplitude suggests increased allocation of attentional resources to the categorization process (Li et al., 2023). Despite the RH group’s poorer behavioral performance, this enhanced occipital activation may reflect an adaptive strategy to manage the cognitive demands of the allocentric task.

In HC group, the uncorrected *P* values for the correlations between perimeter and IES were significant in both egocentric and allocentric conditions. However, these could not be observed after FDR correction (Fig. 5B). This absence of significance may result from the limited statistical power due to the small sample size. The RH group showed no significant correlation between balance function and visuospatial function, whereas the LH group exhibited a positive correlation between these two functions in both egocentric and allocentric conditions. These findings align with previous research showing a relationship between balance and visuospatial abilities, though earlier studies did not differentiate between hemispheric sides (Bergqvist et al., 2023, Robertson et al., 1997, Cohen and Andersen, 2002). Another potential explanation is that, consistent with the interhemispheric competition model, the decrease inhibition over right hemisphere from left damaged hemisphere may amplify the relationship between balance function and visuospatial function in LH group compared with RH and HC groups (Battelli et al., 2017, Hilgetag et al., 2001). Our results provide novel evidence suggesting that the relationship between balance and visuospatial attention is hemispherically dependent.

When the left hemisphere is affected, the right hemisphere plays a crucial role in compensating for both visuospatial attention and balance functions (Peters et al., 2015). This suggests that the positive correlation observed in the LH group reflects the compensatory capacity of the right hemisphere, which integrates visuospatial processing to support balance. In contrast, the absence of this correlation in the RH group may point to a disruption of the integrative mechanisms necessary for linking visuospatial and balance functions. These findings have important implications for developing targeted rehabilitation strategies for individuals post-stroke. For LH stroke survivors, rehabilitation programs might benefit from integrating visuospatial training to enhance balance function, leveraging the positive correlation observed in this group. Conversely, for RH stroke survivors, the lack of correlation suggests that traditional visuospatial training alone may not effectively improve balance. Correlations between ERP and balance performance indicated that enhanced P2 amplitude at the O2 electrode was associated with worse balance performance in the LH group. This could be supported by the previous findings in the literature. The P2 amplitude is understood to reflect the cortical sensorimotor processing linked to postural control, which is affected by the need for a balance response. A decrease in P2 amplitude is typically observed when a balance response is required (Varghese et al., 2017). These findings further confirm the neural mechanism of the right hemisphere in visuospatial processing.

## 5 Limitation

This study has several limitations that should be addressed. First, dynamic balance evaluation, such as gait analysis, was not included. Incorporating such assessments could provide a more comprehensive understanding of how visuospatial attention influences balance during real-world movements. Second, the cross-sectional design of this study limits its ability to establish causal relationships or track changes over time. Longitudinal studies are necessary to determine whether targeted improvements in visuospatial attention can lead to sustained enhancements in balance function, particularly in LH stroke survivors. There needs to be greater attention paid to the differences in timing of enrollment post-stroke that has a significant role in the interpretation of potential mechanisms underlying the observed findings. Third, our study only clarified hemispheric differences in visuospatial function among stroke patients without neglect and severe aphasia. It needs to take caution to generalize our findings to all the stroke population. Future studies should explore the effect of neglect or severe aphasia on the visuospatial capacity by using the subgroup analysis. Fourth, we did not investigate the influence of ipsilesional and contralesionally visual fields on visuospatial attention performance. Future study should examine this factor. Lastly, we could not explore the influence of stroke type, lesion size and location and stroke severity due to the small sample size. The potential influence of these factors should be explored in the future study. Addressing these limitations in future research will provide a deeper and more nuanced understanding of the interplay between visuospatial attention and balance function in different hemispheric stroke patients.

## 6 Conclusion

In this study, LH group exhibited superior balance function and visuospatial attention compared to RH group. Furthermore, a positive correlation between balance function and performance in both egocentric and allocentric reference frame conditions was identified exclusively in the LH group. In addition, reduced P1 amplitude and increased P2 amplitude were found in allocentric reference frame processing among the RH group. These findings revealed the potential lateralized roles of the cerebral hemispheres in integrating balance and visuospatial attention functions in the patients with mild to moderate stroke.

## Funding

The author(s) disclosed receipt of the following financial support for the research, authorship, and/or publication of this article: This research was supported by the Guangdong-Hong Kong Technology and Innovation Cooperation Funding (No. 2023A0505010014), the National Key Research and Development Program of China (No. 2022YFC2009700), the Guangdong Basic and Applied Basic Research Foundation (No. 2024A1515011685), the Medical Scientific Research Foundation of Guangdong Province of China (No. B2023098) and the Program of Guangdong Provincial Clinical Research Center for Rehabilitation Medicine (No. 2023B110003).

## CRediT authorship contribution statement

**Shijue Li:** Writing – original draft, Visualization, Formal analysis, Data curation, Conceptualization. **Kai Li:** Writing – original draft, Investigation, Formal analysis. **Ziyan Huang:** Writing – original draft, Investigation, Formal analysis. **Zhenwen Liang:** Validation, Methodology. **Huaqing Chen:** Validation, Methodology. **Yongping Zheng:** Validation, Methodology. **Chuhuai Wang:** Writing – review & editing, Supervision, Project administration, Funding acquisition. **Qiuhua Yu:** Writing – review & editing, Supervision, Project administration, Methodology, Funding acquisition, Conceptualization. **Minghui Ding:** Writing – review & editing, Supervision, Project administration, Methodology, Funding acquisition, Conceptualization.

## Declaration of competing interest

The authors declare that they have no known competing financial interests or personal relationships that could have appeared to influence the work reported in this paper.

## Data Availability

Data will be made available on request.
