# Supplementary material for: The relationship between balance and visuospatial attention on hemispheric stroke survivors: A study of egocentric and allocentric neural processing
Source: Neuroimage Clin. 2025 Aug 9;48:103861. doi: 10.1016/j.nicl.2025.103861 (PMC12362702; doi:10.1016/j.nicl.2025.103861)
Supplement: Supplementary Data 1 [file mmc1.docx]

**Supplemental Table1.** Comparison of Cognitive Tests among the three groups.

|  | RH  (mean$\pm$SD) | LH  (mean$\pm$SD) | HC  (mean$\pm$SD) |  | Between group comparisons | *P* |
| --- | --- | --- | --- | --- | --- | --- |
| MoCA | 23.18$\pm$4.72 | 23.38$\pm$4.33 | 26.2$2\pm$2.44 |  | RH, LH | 0.885 |
|  |  |  |  |  | RH, HC | 0.026^*^ |
|  |  |  |  |  | LH, HC | 0.040^*^ |
| TMT-A(s) | 74.88$\pm$38.08 | 51.06$\pm$23.63 | 46.94$\pm$21.98 |  | RH, LH | 0.022 ^*^ |
|  |  |  |  |  | RH, HC | 0.006^**^ |
|  |  |  |  |  | LH, HC | 0.679 |
| TMT-B(s) | 113.94$\pm$53.96 | 81.44$\pm$35.98 | 90.78$\pm$62.40 |  | RH, LH | 0.082 |
|  |  |  |  |  | RH, HC | 0.198 |
|  |  |  |  |  | LH, HC | 0.607 |

***P*<0.01. **P*<0.05.

**Supplemental Table2.** Comparison of balance and visual spatial attention parameters among the three groups.

|  | RH  (mean$\pm$SD) | LH  (mean$\pm$SD) | HC  (mean$\pm$SD) |  | Between group comparisons | *p* |
| --- | --- | --- | --- | --- | --- | --- |
| AP velocity(mm/s) | 5.65$\pm$2.12 | 4.59$\pm$2.27 | 5.78$\pm$1.40 |  | RH, LH | 0.128 |
|  |  |  |  |  | RH, HC | 0.844 |
|  |  |  |  |  | LH, HC | 0.084 |
| ML velocity(mm/s) | 4.38$\pm$3.03 | 2.56$\pm$1.67 | 3.83$\pm$1.34 |  | RH, LH | 0.018^*^ |
|  |  |  |  |  | RH, HC | 0.451 |
|  |  |  |  |  | LH, HC | 0.090 |
| Ellipse area(mm^2^） | 179.65$\pm$141.60 | 72.72$\pm$79.03 | 89.78$\pm$49.79 |  | RH, LH | 0.003^**^ |
|  |  |  |  |  | RH, HC | 0.009^**^ |
|  |  |  |  |  | LH, HC | 0.613^*^ |
| Perimeter(mm) | 252.32$\pm$109.69 | 180.78$\pm$94.62 | 204.16$\pm$98.20 |  | RH, LH | 0.034^*^ |
|  |  |  |  |  | RH, HC | 0.026^*^ |
|  |  |  |  |  | LH, HC | 0.967 |

Abbreviation: AP, anterior-posterior; ML, medium-lateral. ***P*<0.01. * *P*<0.05.

**Supplemental Table3**.Comparison of visual attention parameters among the three groups.

|  | RH  (mean$\pm$SD) | LH  (mean$\pm$SD) | HC  (mean$\pm$SD) |  | Between group comparisons | *p* |
| --- | --- | --- | --- | --- | --- | --- |
| EgoRT  (ms) | 942.08$\pm$267.88 | 898.96$\pm$312.98 | 783.63$\pm$346.71 |  | RH, LH | 0.693 |
|  |  |  |  |  | RH, HC | 0.139 |
|  |  |  |  |  | LH, HC | 0.287 |
| EgoACC  (%) | 85.44$\pm$15.28 | 94.45$\pm$7.35 | 95.97$\pm$7.21 |  | RH, LH | 0.019^*^ |
|  |  |  |  |  | RH, HC | 0.005^**^ |
|  |  |  |  |  | LH, HC | 0.679 |
| EgoIES  (ms/%) | 1177.59$\pm$510.72 | 972.55$\pm$411.67 | 840.64$\pm$472.81 |  | RH, LH | 0.215 |
|  |  |  |  |  | RH, HC | 0.038^*^ |
|  |  |  |  |  | LH, HC | 0.416 |
| AlloRT  (ms) | 1245.49$\pm$367.38 | 1027.75$\pm$278.26 | 997.11$\pm$272.03 |  | RH, LH | 0.049^*^ |
|  |  |  |  |  | RH, HC | 0.021^*^ |
|  |  |  |  |  | LH, HC | 0.774 |
| AlloACC  (%) | 84.49$\pm$18.53 | 89.06$\pm$10.02 | 95.53$\pm$6.62 |  | RH, LH | 0.306 |
|  |  |  |  |  | RH, HC | 0.013^*^ |
|  |  |  |  |  | LH, HC | 0.145 |
| AlloIES  (ms/%) | 1651.30$\pm$940.41 | 1174.91$\pm$361.37 | 1062.55$\pm$361.00 |  | RH, LH | 0.032^*^ |
|  |  |  |  |  | RH, HC | 0.007^**^ |
|  |  |  |  |  | LH, HC | 0.599 |

Abbreviation: EgoRT, egocentric reaction time; EgoACC, egocentric accuracy rate; EgoIES, egocentric efficiency score; AlloRT, allocentric reaction time; AlloACC, allocentric accuracy rate; AlloIES, allocentric efficiency score. ***P*<0.01. **P*<0.05.

**Supplemental Table4.** Correlation analysis between the balance function and cortical evoked potential in the LH group in the allocentric reference frame condition.

|  |  | P1 at P8 | P2 at O1 | P2 at O2 |
| --- | --- | --- | --- | --- |
| AP velocity |  |  |  |  |
|  | r | 0.161 | 0.376 | 0.463 |
|  | *P* | 0.583 | 0.185 | 0.096 |
| ML velocity |  |  |  |  |
|  | r | 0.347 | 0.348 | 0.535^*^ |
|  | *P* | 0.225 | 0.223 | 0.049 |
| ellipse area |  |  |  |  |
|  | r | 0.209 | 0.490 | 0.667^**^ |
|  | *P* | 0.474 | 0.076 | 0.009 |
| Perimeter |  |  |  |  |
|  | r | 0.172 | 0.450 | 0.580^*^ |
|  | *P* | 0.556 | 0.106 | 0.030 |

Abbreviation: AP, anterior-posterior; ML, medium-lateral. ** *P*<0.01. * *P*<0.05.
